# Laparoscopic liver resection for liver metastasis of leiomyosarcoma of the thigh: a case report

**DOI:** 10.1186/s40792-022-01400-1

**Published:** 2022-03-21

**Authors:** Shoichi Tsuzaka, Yoh Asahi, Toshiya Kamiyama, Tatsuhiko Kakisaka, Tatsuya Orimo, Akihisa Nagatsu, Takeshi Aiyama, Takeyuki Uebayashi, Hirofumi Kamachi, Masatake Matsuoka, Kento Wakabayashi, Takuya Otsuka, Yoshihiro Matsuno, Akinobu Taketomi

**Affiliations:** 1 Department of Gastroenterological Surgery I, Hokkaido University Hospital, Kita-ku, Kita 15, Nishi 7, Sapporo, Hokkaido 060-8638 Japan; 2 Department of Orthopaedic Surgery, Faculty of Medicine and Graduate School of Medicine, Hokkaido University, Kita-ku, Kita 15, Nishi 7, Sapporo, Hokkaido 060-8638 Japan; 3 Department of Surgical Pathology, Hokkaido University Hospital, Kita-ku, Kita 15, Nishi 7, Sapporo, Hokkaido 060-8638 Japan

**Keywords:** Laparoscopic liver resection, Liver metastasis of leiomyosarcoma, Liver metastasis of leiomyosarcoma of the thigh

## Abstract

**Background:**

Although there is no established treatment strategy for liver metastasis of leiomyosarcoma, liver resection has been reported to be effective in some cases. However, almost all liver resections performed for liver metastasis of primary leiomyosarcoma are reported to be open resections, and there are few reports of liver resection performed by laparoscopy. Here, we report a case of laparoscopic liver resection for liver metastasis of a leiomyosarcoma in the right thigh.

**Case presentation:**

An 80-year-old man was diagnosed with leiomyosarcoma of the right thigh with liver metastasis. The primary tumor was first resected, and he was discharged on the 25th postoperative day. Four months after primary tumor resection, a laparoscopic right posterior sectionectomy was performed. There were no postoperative complications, and the patient was discharged on the 11th postoperative day with a histopathological diagnosis of liver metastasis of leiomyosarcoma and negative resection margins. Currently, 9 months have passed since the resection of the primary tumor, and 5 months have passed since the laparoscopic liver resection; there is no recurrence.

**Conclusions:**

The liver metastasis of leiomyosarcoma was successfully removed, with good short-term outcomes after the laparoscopic liver resection. Laparoscopic liver resection seems to be effective for liver metastasis of leiomyosarcoma, which is characterized by a high recurrence rate after surgery. However, more case studies may be necessary to examine the effectiveness and long-term results of laparoscopic liver resection for the treatment of liver metastasis of leiomyosarcoma.

## Background

Leiomyosarcoma is one of the most common histological types of soft-tissue sarcomas, accounting for 5–10% of all cases [1]. It most commonly occurs in the extremities, particularly the lower extremities, retroperitoneum, abdomen/pelvis, and trunk [2, 3]. It has been estimated that 25–40% of patients with soft-tissue sarcoma develop metastases despite appropriate local treatment [4, 5]. The most common site of metastasis, either during the first recurrence or subsequent disease progression, is the lungs, followed by the liver and soft tissue. Less common sites of metastasis are the bones, soft tissue of the chest wall with the exception of the lung parenchyma, and abdomen or retroperitoneum [2, 3].

There is no established treatment for liver metastasis of leiomyosarcoma, but liver resection has been reported to be effective [4–9]. The recurrence rate of leiomyosarcoma is high, even after surgical resection. However, there are reports that re-liver resection is effective [5]. In most previous surgical studies, open surgery was conducted for liver metastases of primary leiomyosarcomas in the extremities; only one study has reported laparoscopic liver resection as a treatment for metachronous liver metastasis [10]. Herein, we report a case of laparoscopic liver resection for synchronous liver metastasis from a primary leiomyosarcoma of the extremities.

## Case presentation

**Fig. 1:**
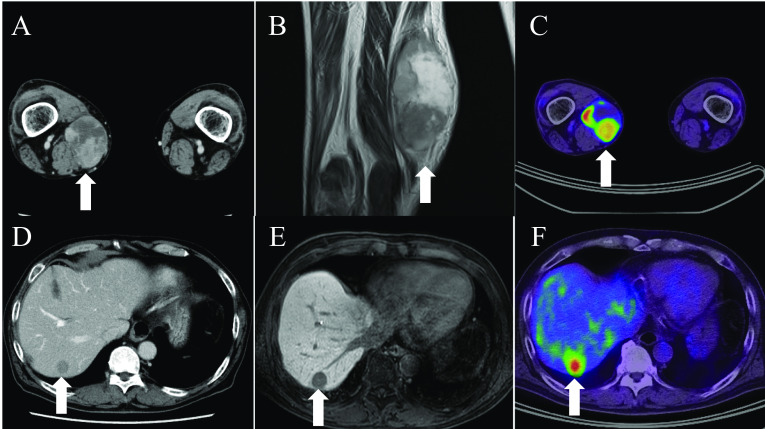
**A** Contrast-enhanced computed tomography (CT) revealing a mass with a heterogeneous contrast effect in the right thigh. **B** T2-weighted magnetic resonance imaging (MRI) showing a solid tumor with high intensity in the right medial thigh, which is suggestive of soft-tissue sarcoma. **C** 18F-fluorodeoxyglucose-positron emission tomography CT indicating fluorodeoxyglucose accumulation with a maximal standardized uptake value of 12.9 in the right thigh. **D** Contrast-enhanced CT revealing a liver tumor with a low contrast effect in the liver S7. **E** Ethoxybenzyl-diethylenetriamine pentaacetic acid (EOB) MRI indicating defective EOB uptake in liver S7. **F** 18F-fluorodeoxyglucose-positron emission tomography CT showing fluorodeoxyglucose accumulation with a maximal standardized uptake value of 12.3 in liver S7

**Fig. 2:**
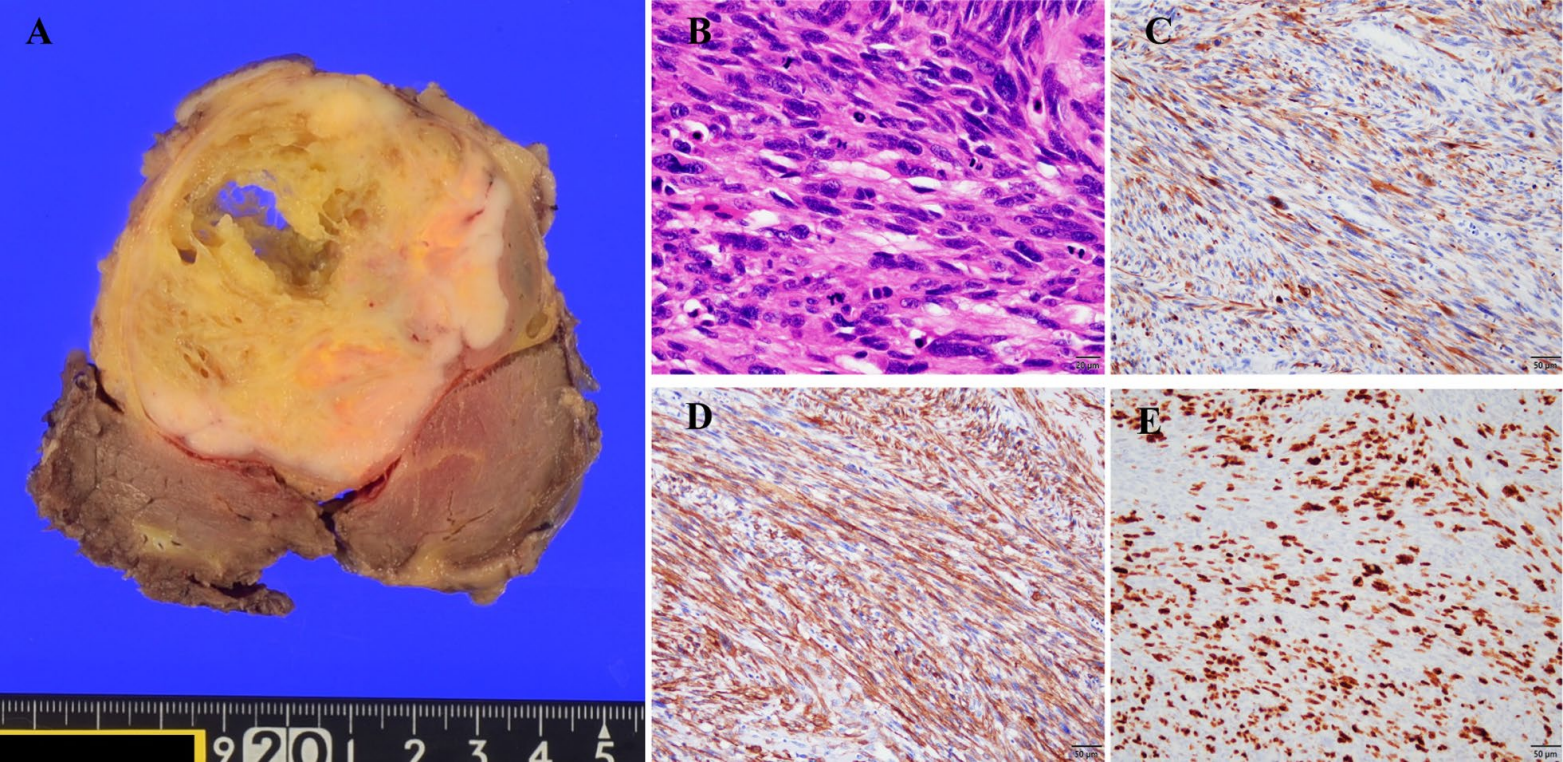
**A** Macroscopic appearance of the primary tumor, measuring 15 × 9.3 × 8.2 cm. **B** Microscopically, the tumor is composed of atypical spindle cells with nuclear pleomorphism and numerous mitotic figures (HE staining, original magnification × 400). **C** Immunohistochemical analysis was positive for desmin (Immunohistochemistry, original magnification × 200). **D** Immunohistochemical analysis was positive for α-SMA (Immunohistochemistry, original magnification × 200). **E** Ki-67 proliferative rate for the primary tumor was 60% (Immunohistochemistry, original magnification × 200)

**Fig. 3:**
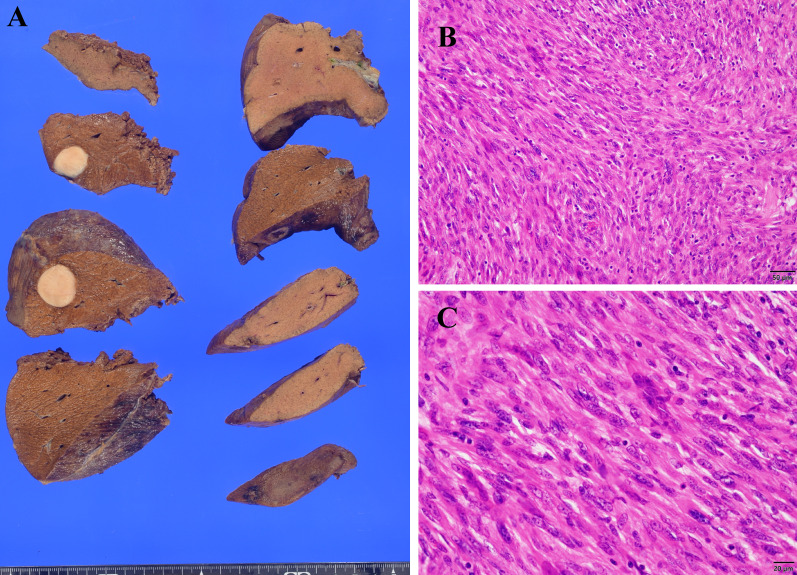
A Macroscopic findings of liver metastasis of the leiomyosarcoma. **A** well circumscribed solitary mass is seen, measuring 21 × 20 × 15 mm. **B,** **C** Microscopically, the morphology is similar to that of the primary tumor (**B** HE staining, original magnification × 200, **C** HE staining, original magnification × 400)

An 80-year-old man presented to the hospital with an enlarged mass in his right thigh. His medical history included hypertension and diabetes mellitus; however, his general condition was good. Upon physical examination, an 18-cm mass was palpated in his right thigh. Laboratory blood test findings were normal. Indocyanine green retention at 15 min was 4.3% and technetium-99 m diethylenetriaminepentaacetic acid galactosyl human serum albumin revealed a blood clearance index (HH15; uptake ratio of the heart at 15 min to that at 3 min) of 0.535 and receptor index (LHL15; uptake ratio of the liver to the liver plus heart at 15 min) of 0.933, indicating good liver function. Whole-body computed tomography (CT) revealed a 25-mm mass with heterogeneous contrast effect in the right thigh and a low-density mass in segment 7 of the liver (S7). T2-weighted magnetic resonance imaging (MRI) revealed a 25-mm solid tumor with a high-intensity signal at the right medial thigh, which was suggestive of a soft-tissue sarcoma. A gadolinium-ethoxybenzyl-diethylenetriamine pentaacetic acid (EOB) MRI revealed decreased EOB uptake in the liver S7. Moreover, 18F-fluorodeoxyglucose-positron emission tomography CT revealed fluorodeoxyglucose accumulation with a maximal standardized uptake value of 12.3 in the liver S7, which was suggestive of synchronous metastasis (Fig. 1). There were no other distant metastatic lesions. A percutaneous needle biopsy was performed from the soft tissue mass of the right thigh, resulting in a histopathological diagnosis of leiomyosarcoma. Subsequently, a CT-guided needle biopsy was performed on the liver mass, resulting in a histopathological diagnosis of liver metastasis of leiomyosarcoma. Resection of the primary tumor was planned to be performed first, followed by liver resection. The right thigh tumor was resected, along with a portion of the vastus medialis. On the 14^th^ postoperative day, antibiotics were administered owing to wound redness and elevated levels of inflammatory markers. Thereafter, the patient’s condition progressed without any problems, and he was discharged on the 25^th^ postoperative day. A histopathological diagnosis of leiomyosarcoma was made based on the presence of atypical spindle cells with numerous mitotic figures [42 mitoses/10 high-power fields] (Fig. 2A) in addition to necrosis in more than 50% of the tumor volume, which resulted in classification as French Federation of Cancer Centers grade III. Immunohistochemical detection of desmin (Fig. 2B) and α-SMA (Fig. 2C) as well as a Ki-67 labeling index of up to 60% (Fig. 2D) confirmed the diagnosis. Four months after primary tumor resection and confirmation that the liver was the sole site of metastasis along with no change in its size based on imaging findings, laparoscopic right posterior sectionectomy was performed. The surgical time was 5 h 14 min, and blood loss volume was 80 mL. There were no postoperative complications, and the patient was discharged on the 11th postoperative day. The histopathological diagnosis was liver metastasis of leiomyosarcoma with negative resection margins (Fig. 3). Currently, 9 months have passed since the resection of the primary tumor, and 5 months have passed since laparoscopic liver resection, and there is no tumor recurrence. We observed the patient through periodic follow-ups.

## Discussion

We performed metachronous laparoscopic liver resection for synchronous liver metastasis of primary leiomyosarcoma of the right thigh. Only one case of laparoscopic liver resection for liver metastasis of leiomyosarcoma was reported previously [10].

Although various treatment modalities have been investigated, liver resection is considered to be an effective treatment for liver metastases derived from leiomyosarcoma. Liver resection for liver metastases derived from leiomyosarcoma is reported to result in a 5-year overall survival (OS) of 12–51.8% and a median survival time (MST) of 11–77 months [4–6, 8, 9, 11–15] (Table 1). Other treatment options are systemic chemotherapy, chemoembolization, radiofrequency ablation, and liver resection [4–6, 8, 9, 16–21]. However, the response rate of liver metastases of leiomyosarcoma to chemotherapy is low, with response durations of only a few weeks to months [22, 23], and chemoembolization is not effective for hypovascular liver metastases such as the present case [24]. For these reasons, liver resection was elected as the most appropriate treatment option for the present case. Partial liver resection and posterior sectionectomy were considered for this case; however, posterior sectionectomy was indicated due to reports that remnant liver ischemia after liver resection can result in a higher recurrence rate and worse survival outcomes in cases of liver metastases from a malignant tumor [25]. At the same time, the patient’s liver function was good enough to tolerate the posterior sectionectomy, which was safely performed as a laparoscopic procedure without any postoperative complications.

**Table 1:** Survival after hepatectomy for metastatic leiomyosarcoma with complete surgical resection in previous reports

Period	References	Cases (n)	MST (months)	OS (%)		
				1-year	3-year	5-year
-1978	[17]	12	11	58	23	12
1982–1996	[8]	15	32	NA	NA	20
1982–2000	[6]	42	39	88	50	30
1993–2003	[5]	27	44	NA	NA	49
1990–2009	[18]	98	72	NA	60	32
1997–2009	[4]	31	24	90.3	48	31.8
2000–2009	[19]	27	NA	NA	NA	46
1998–2013	[21]	21	77	88	54	42
1998–2014	[9]	47	42.1	NA	64.3	48.4
1998–2015	[20]	62	61.6	98.3	67.1	51.8

*NA* not available

In the present case, the patient had simultaneous liver metastases, and went through two-staged surgeries, with liver resection performed 4 months after resection of primary tumor. According to Marudanayagam et al., there was no significant difference in survival outcome after liver resection between patients with synchronous versus metachronous liver metastases from soft-tissue sarcomas [4]. However, the appropriate time for surgery and a comparison of the effectiveness of the one-stage or two-stage surgical approaches for synchronous liver metastases from leiomyosarcomas has not been established and requires further research and accumulation of cases. For other malignancies, there is no clear difference between synchronous and metachronous resection in cases of synchronous colorectal cancer liver metastases [26, 27].

According to previous reports regarding liver resection for liver metastases of leiomyosarcomas, in almost all cases, only open liver resection performed [4–6, 8, 9, 11–13, 28], and only a few cases involving laparoscopic liver resection have been reported [10]. Owing to advances in surgical instruments and understanding, the indications for laparoscopic liver resection are expanding to include a wide range of liver tumors [29], and this method is now practiced in many countries. Several reports suggest that laparoscopic liver resection leads to lower intraoperative blood loss than does open liver resection due to the magnified field of view and reduced bleeding from the hepatic veins because of insufflation pressure and reduced adhesions [30–32]. In addition, it has been reported that postoperative complications such as ascites were less common in laparoscopic liver resection than in open liver resection because of the smaller wound and decreased mobilization associated with laparoscopic surgery compared to those associated with surgery for hepatocellular carcinoma [33, 34]. In hepatocellular carcinoma, laparoscopic liver resection and open liver resection result in similar long-term outcomes; OS and recurrence-free survival have been found to be equivalent between the two methods [32, 35]. In addition, no studies have specifically disagreed with the use of laparoscopic liver resection in malignant liver tumors, such as hepatocellular carcinoma and colorectal cancer liver metastases. Currently, few reports regarding laparoscopic liver resection for liver metastasis of leiomyosarcoma exists; however; the number of reports is expected to increase in the future, and survival outcomes should be thoroughly investigated.

Leiomyosarcoma liver metastasis is characterized by a high recurrence rate of approximately 43.7–88% after surgery [4–6, 28]. Rehders et al. performed re-liver resection in two of four patients with hepatic recurrence after liver resection for liver metastasis of leiomyosarcoma. The MST for patients who underwent re-liver resection was 76 months, while the MST for patients who did not undergo re-liver resection was 26 months. They showed that re-resection of recurrent intrahepatic lesions led to a significantly better prognosis than that with non-re-resection [5]. Moreover, postoperative adhesions are less likely to form after laparoscopic liver resection than after open liver resection due to decreased mobilization during the laparoscopic procedure [36, 37]. The possibility of re-liver resection should be considered for this class of tumor owing to their high rate of recurrence associated with it. Therefore, laparoscopic liver resection, which results in fewer adhesions and a lower rate of complications, seems to be a more effective method for the treatment of liver metastasis of leiomyosarcoma.

## Conclusions

In conclusion, we present the case of a patient in whom the liver metastasis of a leiomyosarcoma was successfully removed by laparoscopic liver resection with good short-term outcomes. Our findings suggest that laparoscopic liver resection is an effective treatment approach for liver metastasis of leiomyosarcoma, which have a high recurrence rate but are effectively treated by re-liver resection. However, because this case had a postoperative observation period of only 7 months, more case studies are warranted to examine the long-term effectiveness of laparoscopic liver resection for the treatment of liver metastasis of leiomyosarcoma.

## Data Availability

Not applicable.

## Abbreviations

CT: Computed tomography
S7: Segment 7
MRI: Magnetic resonance imaging
EOB: Gadolinium-ethoxybenzyl-diethylenetriamine pentaacetic acid
OS: Overall survival
MST: Median survival time
R0: Complete surgical resection
